# Supplementary material for: ALGD-ORB: An improved image feature extraction algorithm with adaptive threshold and local gray difference
Source: PLoS One. 2023 Oct 23;18(10):e0293111. doi: 10.1371/journal.pone.0293111 (PMC10593235; doi:10.1371/journal.pone.0293111)
Supplement: Attachment — Submitted filename: Response to Reviewers.docx [file pone.0293111.s001.docx]

**Original Manuscript ID:** PONE-D-23-24466

**Original Article Title: “**ALGD-ORB: An Improved Image Feature Extraction Algorithm with Adaptive Threshold and Local Gray Difference”

**To:** PLOS ONE Editor

**Re:** Response to reviewers

Dear Editor,

Thank you for allowing a resubmission of our manuscript, with an opportunity to address the academic editor and reviewers’ comments.

We are uploading (a) our point-by-point response to the comments (below) (Response to Reviewers), (b) an updated manuscript with yellow highlighting indicating changes (under “Revised Manuscript with Track Changes*”*), and (c) a clean updated manuscript without highlights (“Manuscript”*).*

Best regards,

<G. Chu> et al.

**Reviewer#1, Concern # 1:** The abstract should be abbreviated in a concise way.

**Author response:** Thank you for your feedback. We apologize for the abstract's length. We have revised the abstract to provide a more concise summary of our work while still conveying its key contributions and significance.

**Author action:** We have shortened the abstract from 204 to 157 words, ensuring the contextualization of the application area. Additionally, we have explicitly mentioned the full name of the ORB algorithm as "Oriented FAST and Rotated BRIEF (ORB)" in the abstract.

**Reviewer#1, Concern # 2:** The research motivation should be strengthened in the introduction. I recommend the authors to restructure the introduction into four subsections: background, current challenges or limits of prior SOTAs, research motivation, main contribution (here the authors may still use original text, but in a different order).

**Author response:** We appreciate the reviewer's suggestions. We agree that the introduction should more explicitly highlight the research motivation, and we have restructured the introduction accordingly, following your recommendations. The introduction now comprises four subsections: 1.1 Background, 1.2 Challenges or Limitations of Current Techniques, 1.3 Research Motivation, and 1.4 Key Contributions. This structure not only facilitates a clearer understanding of the research background and significance for the readers but also explicitly outlines the specific issues and challenges that this study aims to address.

**Author action:** In accordance with the reviewer's advice, we have reorganized the introduction section. Particularly in the "Research Motivation (1.3)" subsection, we have provided detailed descriptions of the specific limitations and challenges of the ORB algorithm that this research seeks to address, along with why these challenges warrant attention. We believe that these modifications not only align with the reviewer's expectations but also enhance the completeness and comprehensibility of the paper.

**Reviewer#1, Concern # 3:** In related works,the authors should highlight what has been previously investigated or what has not been explored? On this basis, the novelty should be summarized by comparing with these listed literatures.

**Author response:** Thank you for your constructive feedback. We appreciate your suggestion to clarify the gaps in existing literature and to explicitly state the novelty of our work in relation to these gaps. We agree that doing so will provide a clearer context for our contributions.

**Author action:** Revised Related Works Section:

In the revised version of the paper, the related works section will be restructured to explicitly highlight the gaps and limitations in existing literature. Each subsection will conclude with a summary of what has been previously investigated and what remains unexplored. This will set the stage for introducing the novelty of our work.

For example, after discussing traditional sparse feature extraction methods like SIFT, SURF, and ORB, we can add:

"Although these traditional algorithms have been foundational in the field, they often suffer from computational inefficiency, sensitivity to noise, and lack of robustness in varying conditions. While some improvements have been made, there is still a significant gap in achieving real-time performance with high accuracy."

Similarly, after discussing deep learning-based methods like LIFT, SuperPoint, and D2-Net, we can add:

"While deep learning-based methods have shown promise in terms of performance, they often require large amounts of labeled data and computational resources, limiting their applicability in resource-constrained environments. Furthermore, the integration of these methods into existing SLAM systems remains an open challenge."

Summary of Novelty:

Finally, the section will conclude with a summary paragraph that explicitly states the novelty of our work in comparison to the listed literatures:

"Our work addresses these gaps by introducing an adaptive thresholding method for feature point detection and a novel binary descriptor formation technique. Unlike traditional methods, our approach considers local pixel intensity variations, making it more robust under varying lighting conditions. Additionally, our feature descriptor is less sensitive to noise, addressing a common limitation in existing binary descriptors. This work represents a significant step towards achieving real-time, accurate, and robust feature point detection and description, particularly in the context of SLAM systems."

By making these changes, we aim to clearly articulate the unique contributions of our research in the context of existing literature. Thank you for helping us improve the quality of our paper.

**Reviewer#1, Concern # 4:** The confusion matrices should be provided.

**Author response:** Author response: Thank you for your feedback. We appreciate your interest in our work. However, in the context of image feature extraction and matching algorithms, confusion matrices are typically not employed as evaluation metrics. Confusion matrices are primarily utilized for assessing the performance of classification algorithms, which involve categorizing instances into different classes. In the case of our proposed ALGD-ORB algorithm, which focuses on image feature extraction and matching, we primarily utilize other relevant evaluation metrics such as feature point distribution uniformity, accuracy, RMSE and real-time performance. These metrics provide insights into the effectiveness and efficiency of our algorithm in terms of its intended purpose. If there are any other specific evaluation metrics or aspects you would like us to address, or if there are any further inquiries you have, please feel free to let us know.

**Author action:**

**Reviewer#1, Concern # 5:** What are units in Table 5? The computational complexity needs to be analyzed or compared.

**Author response:** We apologize for any oversight. In Table 5, the units of measurement for the mean operation time are seconds. As for the computational complexity, we appreciate the reviewer's point about the need for a detailed analysis or comparison.

**Author action:** We will make sure to clarify the units of measurement in Table 5 to indicate that the mean operation time is presented in seconds. Furthermore, we have made the necessary changes to Table 4 to include the units of measurement. The units for Table 4 have been updated to pixels. Thank you for your feedback, and we are grateful for your thorough review. While recognizing the absence of a formal analysis of computational complexity, it's important to note that due to time constraints, a comprehensive assessment was not carried out in this study. Nevertheless, addressing this aspect remains a crucial endeavor for future research.

**Reviewer#1, Concern # 6:** I also recommend the authors to refer to several recent machine learning literatures.

a. 10.1109/TKDE.2023.3277839

b. 10.1109/JBHI.2022.3193148

c. 10.1016/j.knosys.2023.110789

d. 10.1109/TETCI.2021.3136642

**Author response:** After thoroughly examining the abstracts of these papers, it appears that the topics covered are primarily focused on aspects related to human activity recognition, deep learning, and contrastive learning. While these papers provide valuable insights into machine learning techniques, they are not directly related to our current research focus on image feature extraction and matching. As such, we believe that citing these references might not align closely with the specific scope of our work. However, we greatly appreciate your suggestions and would be open to considering any additional references that are more closely aligned with our research on image matching and feature extraction. If you have any other specific literature suggestions or if you would like us to elaborate further on our decision, please let us know. We remain committed to enhancing the quality and relevance of our manuscript based on your valuable feedback.

**Author action:** We have thoroughly reviewed the suggested literature references (a. DOI: 10.1109/TKDE.2023.3277839, b. DOI: 10.1109/JBHI.2022.3193148, c. DOI: 10.1016/j.knosys.2023.110789, d. DOI: 10.1109/TETCI.2021.3136642). While we acknowledge their significance in the field of machine learning and human activity recognition, we have decided not to cite them in our paper as their subject matter does not closely align with our research focus on image feature extraction and matching. We are grateful for your thoughtful recommendations and are committed to enhancing the relevancy and quality of our manuscript based on your feedback. If you have any further recommendations or inquiries, please feel free to let us know.

**Reviewer#2, Concern # 1:** Abstract - Authors should consider starting the abstract with a brief contextualization of the application area, and its importance, of the proposed work. Please, provide the full name of the ORB algorithm.

**Author response:** Thank you for your suggestion. We appreciate your input and agree that contextualizing the application area. Additionally, we will ensure to provide the full name of the ORB algorithm as "Oriented FAST and Rotated BRIEF (ORB)" in the abstract.

**Author action:** We have revised the abstract to begin with a brief contextualization of the application area and its significance. We have also included the full name of the ORB algorithm as "Oriented FAST and Rotated BRIEF (ORB)" in the abstract.

**Reviewer#2, Concern # 2:** Some citations are not well formatted (for example: lines 33, 36 and 37).

**Author response:** After a thorough review of the text in lines 33, 36, and 37, we have confirmed that the citations are correctly and appropriately formatted. The citations adhere to the required academic citation style and are consistent with the references provided in the bibliography. We appreciate your attention to detail and thank you for highlighting this matter. Additionally, if my interpretation of your concern is not accurate, I kindly request further clarification. It's important for us to fully understand your feedback to ensure that we address any potential issues or misunderstandings. Your guidance is invaluable, and we are committed to refining our manuscript in accordance with your feedback.

**Author action:** If the concern pertains to the formatting of references, we will revise the manuscript to ensure it aligns with the style requirements of PLOS ONE. Our goal is to address any potential formatting issues and ensure that the manuscript meets the publication standards. Additionally, we have utilized LaTeX and BibTeX to meticulously organize and format the references in our paper. This ensures that all citations are consistent and adhere to academic standards. Thank you for your guidance, and we are committed to making the necessary adjustments to enhance the quality of our submission.

**Reviewer#2, Concern # 3:** From line 46 to line 100, the text seems to be the cover letter.

**Author response:** I appreciate your observation and apologize for the oversight. Upon reviewing your feedback, I acknowledge that I inadvertently included the content of a cover letter in the manuscript. This was an unintended mistake on my part.

**Author action:** I have promptly taken action to rectify this issue by removing the mentioned text from the manuscript. I apologize for any inconvenience caused and thank you for bringing this to my attention.

**Reviewer#2, Concern # 4:** In the Introduction section, the authors present a somewhat confusing text, where some of their statements must be carefully reviewed, as they present some inaccuracies or lack foundation. For example:

Lines 100 to 102 - It is true that SIFT is a robust technique against scale changes and rotations. However, its effectiveness decreases in the presence of noise or when dealing with images with complex backgrounds.

Lines 107 to 110 - SURF overcome the SIFT limitation of operating in high-dimensional contexts, being also robust to lighting variations. But it is important to notice that this advantage entails a disadvantage compared to SIFT, since its performance in resource calculation is lower. Furthermore, SURF is not rotation invariant. Additionally, when the authors claim that the SURF algorithm allows real-time execution, it is important to substantiate because, as far as I know, the computational complexity of the SURF algorithm limits its application to real-time systems.

Line 130 - The text "... at the ICCV (IEEE International Conference on Computer Vision) in 2001, ..." is not needed.

Lines 134 to 135 - The sentence "It is about 100 times faster than SIFT algorithm and 10 times faster than SURF algorithm." must be sustained in references.

Lines 225 to 227 - Please, revise the sentence "Mur-Arta [20,21] employed a fixed double-threshold FAST method in the initial..."

Line 253 - When using the acronym oFAST for the first time, the name should be written in full.

Line 254 - When using the acronym rBRIEF for the first time, the name should be written in full.

**Author response:** Thank you for your thorough review and valuable feedback on our manuscript. We greatly appreciate your insights, and we have carefully addressed the issues you raised.

**Author** **action:** Here is our response to each of your concerns:

(1). In the introduction section, we have revised the relevant text to eliminate any potential inaccuracies and have included additional supporting information to enhance clarity and accuracy.
(2). Regarding the description of SIFT and SURF's performance and limitations, we have made adjustments and explicitly highlighted potential computational constraints of the SURF algorithm for real-time applications.
(3). We have removed the reference to "ICC (IEEE International Conference on Computer Vision)" as per your suggestion to eliminate unnecessary information.
(4). Concerning the algorithm speed comparison we mentioned, we have added relevant references to support our statements.
(5). We have revised the description of Mur-Arta's approach to accurately portray their method.
(6). We have provided the full names of oFAST and rBRIEF when using their acronyms for the first time in the text.

We believe these revisions adequately address your concerns and contribute to the overall quality and clarity of the manuscript. Once again, we appreciate your review and valuable suggestions. If there are any other aspects you would like to discuss or further adjustments you deem necessary, please feel free to let us know. We are more than willing to make further modifications as needed.

**Reviewer#2, Concern # 5:** Equation 10 - The equation represents the formula for calculating the threshold value for each grid of the image, so that I_i_max, I_i_min and the largest/smaller gray levels will be the values relative to each grid of the image and not to the whole image.

**Author response:** Thank you for your guidance. We appreciate your attention to detail.

**Author action:** Based on your suggestion, we will make the following modification to the original text:

Original: " where and are the- largest gray value and the- smallest gray value in the image, respectively;"

Modification: " where and are the- largest gray value and the- smallest gray value within each grid of the image, respectively;"

We believe this change accurately reflects the intended meaning and addresses your concern regarding the interpretation of the values in relation to each grid within the image. Thank you for helping us improve the clarity of our work.

**Reviewer#2, Concern # 6:** **Line 462 - Please, correct the typo.**

**Author response:** We have reviewed and addressed the concern regarding the potential typo on line 462.

**Author action:** We have corrected the typo on line 462 as indicated by changing "non-floating-point" to "non-floating point." Additionally, if my interpretation of your concern is not accurate, I kindly request further clarification.

**Reviewer#2, Concern # 7:** Lines 714 to 716 - Considering the values in table 5, I do not understand the statement "These results highlight the outstanding runtime efficiency of the ALGD-ORB algorithm on the Oxford dataset, making it a promising choice, particularly in resource-constrained scenarios."

**Author response:** We appreciate the reviewer's concern about the statement regarding ALGD-ORB's runtime efficiency. The reviewer's confusion may stem from the unclear association between the data in Table 5 and the statement "outstanding runtime efficiency." ALGD-ORB does not have a significant advantage over other algorithms in terms of runtime, so this claim may need further clarification or modification.

**Author action:** To address the reviewer's concern, we will revise the relevant sentence in the paper. The original statement will be changed to: "The ALGD-ORB algorithm provides a balanced performance in terms of runtime and feature extraction capabilities, making it a suitable choice for a variety of applications, especially in resource-constrained scenarios where GPU acceleration is not available."

**Reviewer#2, Concern # 8:** Table 5 - What units of measurement are used?

**Author response:** We apologize for any oversight. In Table 5, the units of measurement for the mean operation time are seconds.

**Author action:** We will make sure to clarify the units of measurement in Table 5 to indicate that the mean operation time is presented in seconds. Furthermore, we have made the necessary changes to Table 4 to include the units of measurement. The units for Table 4 have been updated to pixels. Thank you for your feedback, and we are grateful for your thorough review.

**Reviewer#2, Concern # 9:** The authors compare the proposed algorithm with methods proposed some time ago. There are some recent algorithms where the focus is on improving the ORB algorithm, with which I think it would be positive to compare the work of the authors. This comparative study with more recently proposed approaches would help to understand more clearly the current contribution of the proposed work, as well as its possible innovation. Most references are old. As far as I could see, there is only one reference from 2023, with almost all the rest being earlier than 2020. Authors should consider more recent work.

**Author response:** Thank you for your insightful comments regarding the inclusion of more recent literature for comparison, especially those focusing on improvements to the ORB algorithm.

Why Few 2023 References:

The primary reason for the limited inclusion of 2023 references is that the foundational ideas and methodologies for this paper were conceived in 2022. During that period, we conducted a comprehensive review of the most recent and relevant literature available.

Inclusion of 2023 Literature:

To address your concern, we have included references to three significant 2023 papers in the "Current Challenges or Limits of Prior SOTAs" section:

(1). Wang et al. [33] with FeatureBooster, focusing on keypoint descriptor enhancement.
(2). Wang and Liu [34] who developed SSG-Net for multimodal image registration.
(3). Zhu et al. [38] who proposed an improved ORB-RANSAC algorithm for UAV remote sensing image registration.

While these papers are cited to acknowledge the latest advancements in the field, we have not conducted experimental comparisons with these methods due to the timeline of our research conception and the scope of this paper.

**Author action:** We have updated the manuscript to include these recent works in the literature review section to provide a more comprehensive background. However, it's important to note that these works are cited for contextual understan[1]ding and not for direct experimental comparison with our proposed method.

We hope this clarifies your concerns[2] and thank you for bringing this to our attention.[3]

1.	Hutchison D, Kanade T, Kittler J, Kleinberg JM, Mattern F, Mitchell JC, et al. BRIEF: Binary Robust Independent Elementary Features. In: Daniilidis K, Maragos P, Paragios N, editors. Computer Vision – ECCV 2010. Berlin, Heidelberg: Springer Berlin Heidelberg; 2010. pp. 778–792. doi:10.1007/978-3-642-15561-1_56

2.	Jong Hyuk Kim, Salah Sukkarieh. Recasting SLAM - Towards Improving Efficiency and Platform Independency. In: Paolo Dario, Raja Chatila, editors. Robotics Research The Eleventh International Symposium. Berlin, Heidelberg: Springer; 2005. pp. 399–408. doi:10.1007/11008941_43

3.	Lei Y, Yu Z, Gong Y. An Improved ORB Algorithm of Extracting and Matching Features. IJSIP. 2015;8: 117–126. doi:10.14257/ijsip.2015.8.5.12
